# A Continuous Aerobic Resistance Exercise Protocol for Concussion Rehabilitation Delivered Remotely via a Mobile App: Feasibility Study

**DOI:** 10.2196/45321

**Published:** 2023-06-19

**Authors:** Michael G Hutchison, Alex P Di Battista, Matthew M Loenhart

**Affiliations:** 1 Goldring Centre for High Performance Sport University of Toronto Toronto, ON Canada; 2 David L MacIntosh Sport Medicine Clinic Faculty of Kinesiology and Physical Education University of Toronto Toronto, ON Canada; 3 Faculty of Kinesiology and Physical Education University of Toronto Toronto, ON Canada; 4 Defence Research and Development Canada Toronto Research Centre Toronto, ON Canada

**Keywords:** Bayesian modeling, concussion, exercise rehabilitation, mobile health, mild traumatic brain injury, persisting symptoms

## Abstract

**Background:**

In recent years, several studies have consistently reported the beneficial effects of aerobic exercise in alleviating symptoms following concussion. However, exercise modality recommendations by practitioners are often limited to traditional exercise equipment (eg, treadmills and stationary bikes). Advances in digital technologies may help to overcome this limitation, as mobile apps can now provide users with high-quality instructional videos, programs, and monitoring capabilities using alternative modalities such as resistance exercises. Mobile technologies are also rapidly expanding to deliver and complement in-person clinical care. Thus, it is imperative to evaluate this emerging technology on its feasibility, safety, and clinical utility for concussion care.

**Objective:**

The objective of the study was to determine the feasibility of a mobile app to deliver a resistance exercise protocol with minimal equipment to individuals following concussion. Feasibility was defined by retention, adverse events, and achievement of a target heart rate (HR) of 60%±5% (age-adjusted percentage of max: 220 – age). HR data were collected using an Apple Watch, Series 6. Symptoms were evaluated before and after 3 exercise sessions.

**Methods:**

A 2-week, prospective, single-arm pilot study was conducted on 21 adults diagnosed with a concussion. Users were provided a continuous aerobic resistance exercise (CARE) protocol through a mobile app.

**Results:**

A total of 18 participants (14 female and 4 male) completed a 3-session exercise plan. The median age-adjusted percent of HR max for session 1 was 55.5% (IQR 49%-63%), 58.1% (IQR 50.8%-65.2%) for session 2, and 57.4% (IQR 49.5%-64.7%) for session 3. Individual median HR% across all sessions ranged from 46.9% to 67.4%; furthermore, 10 participants (55.5%) had a total mean HR% within the target HR%, 7 participants had a mean HR% below 55%, and 1 participant had a mean HR% above 65%. In addition, adherence to the plan resulted in a decrease in reported symptom burden with 94% posterior probability.

**Conclusions:**

Following concussion, a CARE protocol delivered through a mobile app resulted in no adverse effects with 14% (n=3/21) attrition over 3 sessions. CARE was successful in achieving an aerobic exercise intensity of 55%-65% of age-adjusted maximum HR in the majority of participants and resulted in a decrease in reported symptom burden. The potential for this platform in concussion rehabilitation warrants further investigation. Future studies are needed to assess the use of this technology throughout concussion recovery in both individuals with acute concussion, and those with persistent symptoms.

## Introduction

Advising physical and cognitive *rest* until symptom-free is an outdated management approach for concussion. The classic *rest* approach was well-intended to limit the risk of reinjury or additional injuries, ease discomfort from symptoms, and possibly promote recovery by decreasing energy demands on the brain. However, this approach can lead to feelings of isolation, creating fear-avoidance behaviors in some individuals that contribute to a self-perpetuating cycle of symptoms [1,2]. Although *rest s*till has a role in acute management following suspected concussion, it should be limited to 24-48 hours [3]. Thus, there has been a transition in the cornerstone management principle for concussion from *rest* to *active* rehabilitation.

Researchers have consistently reported the beneficial effects of aerobic exercise to assist in alleviating symptoms following concussion [4-7]. Since the initial research that reported on the therapeutic benefits of aerobic exercise in those with persistent symptoms [8-10], there has been corroborating evidence for the use of aerobic activity early following injury [11-16]. Some researchers have used graded aerobic exercise testing to create an individualized subsymptom exercise program [11-13,15-17], while others have used age-predicted maximal heart rate (HR) percentages to guide the reintroduction of aerobic exercise [14,18]. To date, the advised exercise prescription is limited to stationary bikes or treadmills.

Other forms of exercise, such as resistance training, may provide an alternative solution for exercise reintroduction with minimal equipment and can be completed remotely or to complement in-person therapy. However, resistance training has not been considered as a viable solution for concussion management [19]. This is largely due to concerns of pronounced alterations in blood pressure with heavy loads, exertion to fatigue or failure, rapid head acceleration, and sensorimotor overload because of complex movement patterns.

Apprehensions to resistance exercise following a concussion could be alleviated with the appropriate exercise prescriptions. That is, it may be reasonable to reintroduce physical movement to create a physiological demand comparable to aerobic exercise, albeit limiting head acceleration, and bracing to increase intraabdominal pressure demand. Given this, we developed a continuous aerobic resistance exercise (CARE) protocol to reintroduce exercise following concussion. CARE seeks to impose a pseudocontinuous or steady-state response by employing a combination of resistance exercises with cardiovascular and musculoskeletal demands with minimum rest. The exercises incorporated in the CARE protocol require participants to be in a static body orientation or perform head accelerations only in the up or down axis; the goal is to create a demand similar to steady-state cycling or treadmill activity.

Resistance exercise programs require greater instruction and cues than those with treadmills and stationary bikes; however, mobile health apps help to overcome this barrier. Mobile tools are ubiquitous, have excellent computational capabilities, and are commonly carried by the person [20,21]. As a result, this platform is well-suited for high-quality instructional videos and programs. The willingness of people to adopt and use mobile health technologies has accelerated in recent years, and solutions to complement in-person health care or address issues of accessibility to health care will continue to be necessary.

Given that we are using a novel modality for individuals recovering from a concussion delivered through a mobile app platform, our initial investigation sought to evaluate the feasibility of the protocol. Specifically, we were interested in evaluating (1) adverse events and retention among users across a 3-session plan, and (2) whether users could achieve a target HR during exercise sessions. We also measured symptoms before and after the protocol. We hypothesized that each CARE session would be completed by all participants and would elicit HRs of 55% (±5) of participants’ age-adjusted maximum HR. We also hypothesized that participants’ symptoms would decrease following the 3-session plan.

## Methods

### Study Design

This study was part of a 2-week, community-based, prospective single-arm pilot study in which adults older than 18 years were prescribed an active, home rehabilitation program (accessed by the Rhea Health app). A convenience sample of 26 individuals diagnosed with a concussion by a physician were recruited through flyers, word-of-mouth, and social media platforms (eg, Twitter). Before the study, participants were screened by a research assistant confirming concussion diagnosis, at least 18 years of age, and access to an iPhone. Individuals with co-morbid injuries (ie, musculoskeletal or soft-tissue injuries and vestibular disorders) or neurological conditions that prevented them from physical activity were excluded from the study. Because of the COVID-19 pandemic and the resultant inability to complete in-person research at our institution, participants were enrolled in the study remotely through Zoom. Study enrollment included documenting relevant medical history, mechanism of injury, initial clinical presentation and current symptoms, a physical activity assessment questionnaire reporting the nature and duration of everyday exercise involvement, and satisfaction with current exercise level. Participants were provided (via mail) with the Rhea Health Inc mobile application on their iPhone and Apple Watch Series 6 (Apple Inc). The exercise prescription on the Rhea Health app was determined by days post concussion and the symptom status of the individual.

### Ethics Approval

This study was approved by the Health Sciences Research Ethics Board, University of Toronto (#39143). To ensure that all potential participants were fully aware of the research study, a research team member provided them with an information sheet outlining eligibility and the study's purpose, methods, and possible risks. Upon confirming eligibility, a videoconference through Zoom was completed to address any questions with respect to the study before consenting to participate. Potential participants were reminded that their choice to participate was entirely voluntary and that they could withdraw from the study at any point without any negative repercussions. Informed consent was obtained by reviewing and signing an electronic document outlining the purpose, procedures, and potential risks of participation in this study; the signed form was submitted using REDCap (Research Electronic Data Capture) electronic data capture tools hosted at the University of Toronto. Data captured through the mobile app and Apple Watch was stored on Amazon Web Servers DynamoDB database in Montreal, Quebec, Canada, using Amazon Web Servers proprietary encryption and a restricted access system to keep data secure. Participants’ emails and passwords were required for accessing the mobile app and syncing Apple Watch data to their Apple Health; however, personally identifiable information was stored in a separate database table and not required for data analysis. Upon study completion, participants received CAD $50 (US $37.16) as compensation for participation and were entered into a lottery for an Apple Watch Series 6.

### Participants

We examined the feasibility of providing the CARE protocol among symptomatic individuals. Of the 26 participants, 21 were provided with the CARE protocol as part of their rehabilitation prescription; the 5 participants excluded from analyses were not provided the CARE protocol, either because they were classified as having a symptom burden too high for aerobic activity, or were too early.

**Figure 1:**
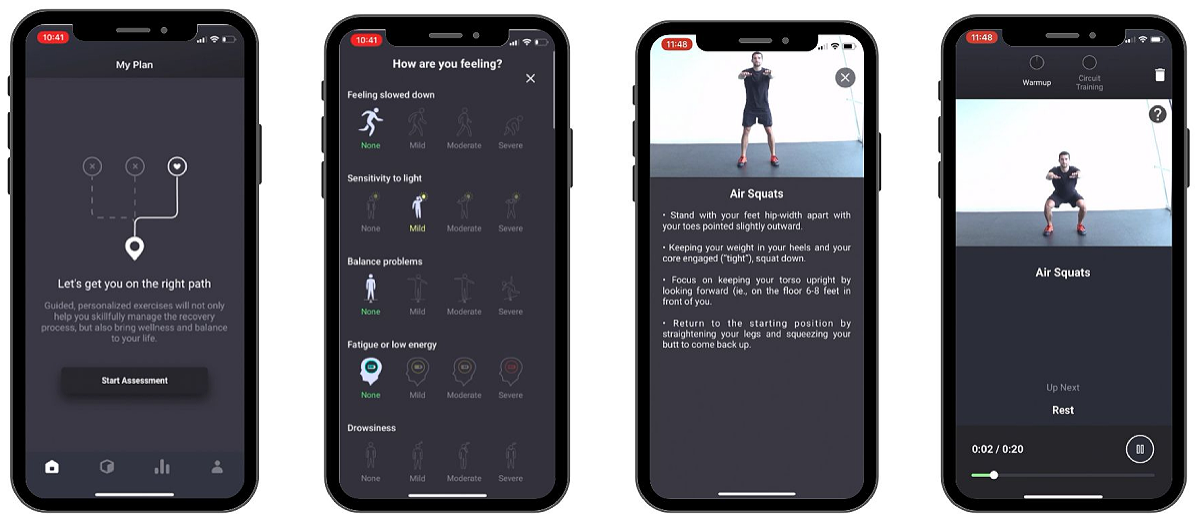
Continuous aerobic resistance exercise (CARE) protocol.

The CARE protocol was delivered through Rhea Health’s mobile app. The mobile app was created to help people recover from concussion using resistance exercises with minimal equipment. Users are provided with instructional videos of exercises to guide them through their rehabilitation process. The app begins when users create an account and provide basic information about their concussion, such as the date of injury and the severity of their symptoms. From there, the app provides users with a personalized exercise program based on their individual needs and abilities. Each exercise is accompanied by clear instructions and visual demonstrations to ensure that users perform the exercises correctly and safely (see Figure 1, screenshots of Rhea’s mobile app). The protocol was devised through collaboration among clinicians, researchers, and strength and conditioning team members at the University of Toronto. This process considered the unique constraints of concussion patients in addition to the principles of strength and conditioning. In the process of identifying exercises, demands on head acceleration, intraabdominal pressure, movement complexity (coordination and balance), and movement speed were considered. Hence, the consensus protocol involved a combination of exercises with limited head acceleration and movement in a single plane (eg, air squats, marching, shoulder press, and mountain climbers). The CARE protocol consisted of 3 sets of a 6-minute exercise circuit for a total session duration of 18 minutes.

### Retention and Adverse Events

Retention was determined by the proportion of participants who completed the 3 CARE sessions. Participants were asked to inform a member of the research staff of any major adverse events (eg, injuries and health concerns) experienced while using the mobile app. The worsening of symptoms resulting in the inability to complete the intervention was categorized as a minor adverse effect.

### Heart Rate

The Apple Watch Series 6 is a wrist-worn smart device (weight range 26.7-52.8 g) that includes a retina organic light-emitting diode display (size 38 or 42 mm), triaxial accelerometer (up to 16 g-forces), triaxial gyroscope, barometric altimeter, optical heart sensor, and global positioning system. This device is water-resistant (up to 50 m) and has up to 18 hours of battery life. It is advertised to monitor HR in real-time using the optical heart sensor. The watch was placed on the dominant wrist of the participants following the manufacturer’s recommendations and connected to an accompanied iPhone throughout the study.

### Symptoms

Participant symptoms were assessed using the symptom checklist included in the mobile application. Because of form factor restrictions of the mobile app, we modified the 22-item postconcussion symptom scale [22] from a 7-point to a 4-point Likert scale (none, mild, moderate, and severe). Symptom severity was calculated by summing the rated score for each symptom up to a maximum of 88. In addition, participants rated any potential change in symptoms following each CARE exercise session (ie, same, better, and worse).

### Statistical Analysis

The objective of the study was to determine the number of participants who achieved the prescribed mean target HR% (±5%) at each session of the 3-session exercise plan. Basic measures of central tendency (mean and median) and spread (SD and IQR) were used to evaluate this objective. Proportions were used to describe the number of participants or sessions that fell within the target HR% range, as well as to evaluate participant session completion. To facilitate group comparisons and compare HR% variance between and within users, and between sessions, a multilevel linear model was employed: mean HR% was modeled with a varying intercept that included an adaptive prior to provide a partially pooled estimate of the mean average HR% for all participants while also providing estimates of the mean HR% at the individual participant level. For differences in symptoms pre- versus postprotocol, an intercept only linear model was used. For between-group differences in HR%, symptoms, and characteristics (ie, concussion history vs no concussion history, history of headache vs no history of headache, etc), linear models with indexed intercepts were employed to facilitate the creation of posterior contrasts. Linear models were also used to evaluate the relationship between HR% and continuous variables (age, individual symptom differences before vs after the protocol). All models employed weakly regularizing priors that had an *a priori* skepticism of group differences and nonzero coefficient weightings. Priors for regression coefficient parameters were of the form α/*β* ~ Normal(0,1), whereas the exponential distribution was used to model the variance parameter σ ~ Exponential(1). Prior to sampling, continuous variables were *z* transformed.

All models were fitted using the Hamiltonian Monte Carlo engine Stan [23] through R [24] and the RStudio Integrated Development Environment [25]. All models ran across 4 chains at 2000 iterations per chain. Convergence was measured using an updated Gelman-Rubin convergence test, with all chains converging to <1.01 [26]. The R package “rethinking” [27] was used to interface between RStudio and RStan [28]. Plots were created using the “ggplot2” [29], “bayesplot” [30,31], and tidybayes [32] packages. Tables were created using the “gt” [33] and “gtsummary” [34] packages.

## Results

### Participant Characteristics

A total of 21 participants were enrolled in the study and prescribed CARE. The participants' median age was 32.3 years (IQR 27.7-38.3) years, they were predominantly female (n=17, 81%), the majority had a prior history of concussion (n=10, 59%), 43% (n=9) had a history of depression, and fewer than half of them were following an exercise program at the time of the study (n=6, 35%). Although only a single participant was a high-performance athlete (n=1, 5.9%), the most common mechanism of concussion was sport or physical activity (n=9, 43%). Only a single participant began using the app in the acute phase of injury (within 1 day); the median time from injury to study enrollment was 85.5 days, with a wide IQR spanning 25.8-618.5 days. See Table 1 for participant and concussion characteristics.

**Table 1:** Participant demographics and concussion characteristics. Due to missing values, percentage calculations may vary.

Characteristic		Participants (N=21)
Age (years), median (IQR)		32.3 (27.7-38.3)
**Sex, n (%)**		
	Female	17 (81)
	Male	4 (19)
History of concussion, n (%)		10 (59)
High performance athlete, n (%)		1 (5.9)
Follow exercise program, n (%)		6 (35)
History of headaches, n (%)		6 (35)
History of ADHD,^a^ n (%)		1 (5.9)
History of depression, n (%)		9 (53)
**Mechanism, n (%)**		
	Sport/PA^b^	9 (43)
	MVC^c^	4 (19)
	Work	2 (9.5)
	Fall	2 (9.5)
	Other	4 (19)
**Time of symptom onset,** **n (%)**		
	Immediate	8 (38)
	Delayed	13 (62)
**Loss of consciousness, n (%)**		
	Yes	1 (5.6)
	No	15 (83)
	Not sure	2 (11)
	Time since injury (days), median (IQR)	85.5 (25.8-618.5)
**Symptom trend since injury, n (%)**		
	No change	1 (5.9)
	Steadily better	7 (41)
	Steadily worse	1 (5.9)
	Worse, then better	1 (5.9)
	Better, then worse	7 (41)

^a^ADHD: attention-deficit/hyperactivity disorder.

^b^PA: physical activity.

^c^MVC: motor vehicle collision.

### Retention and Adverse Events

No adverse events were reported during the 3-session CARE protocol. Of the 21 participants who enrolled in the study, 18 completed the 3 sessions (18/21=85.7%). Two participants were uncomfortable using the technology required, whereas one participant reported being too busy to initiate the protocol.

### Heart Rate

**Figure 2:**
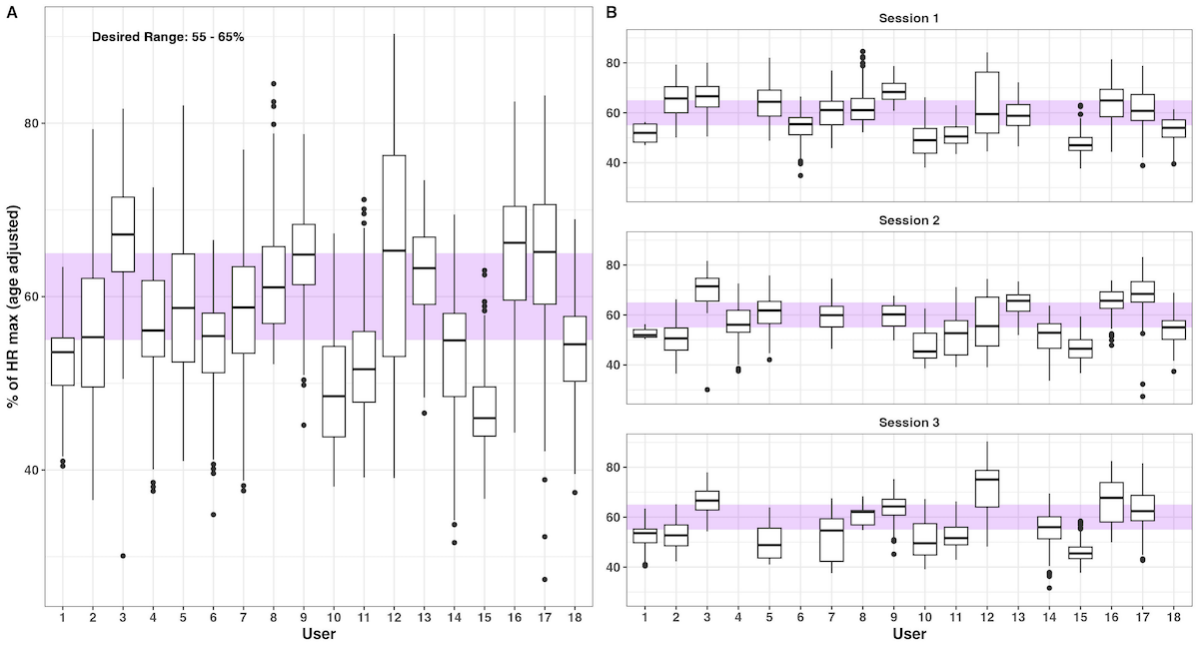
Heart rate data by user throughout the CARE protocol. Box and whisker plots show the median, IQR, and ±1.5 × IQR (whiskers) of participant heart rate data, expressed as a percentage of their age-adjusted heart rate (HR) max, aggregated across all sessions (A) or by individual session (B). The purple band represents the range of the desired target HR%: 55%-65%. CARE: continuous aerobic resistance exercise.

Of the 18 participants who completed the protocol, HR data were captured for all sessions on 12 participants, for 2 sessions on 4 participants, and for a single session on 2 participants. The average session length was 22.4 (IQR 21.25-25) minutes. On average, it took participants a median of 6.5 (IQR 5-9) days to complete the CARE protocol. The median HR across all users in each of the 3 sessions was within the target range (55%-65% of age-adjusted HR max). The mean age-adjusted % of HR max for session 1 was 55.5% (IQR 49%-63%), 58.1% (IQR 50.8%-65.2%) for session 2, and 57.4% (IQR 49.5%-64.7%) for session 3; there were no appreciable differences in the average group-level HR% between sessions (Figure S1 of Multimedia Appendix 1). Individual participants’ median HR% across all sessions ranged from 46.9% to 67.4%; ten participants (55.5%) had a median HR% within the target HR%, 7 participants had a median HR% below 55%, and one participant had a median HR% above 65%. At the session level, of the 46 individual sessions completed by the 18 participants, 19 sessions (41.3%) were within the target HR% range, 8 sessions (17.4%) were above 65%, and 19 sessions (41.3%) were below 55%. HR% varied similarly between and within users across the plan; the average estimated SDs were 6.54% (90% compatibility interval [CI] 4.9%-8.7%) and 7.3% (90% CI 7.2%-7.4%), respectively, resulting in an intraclass correlation of 0.47 (90% CI 0.46-0.6). See Figure 2 for a visual depiction of HR% values within and between users, across all sessions.

**Figure 3:**
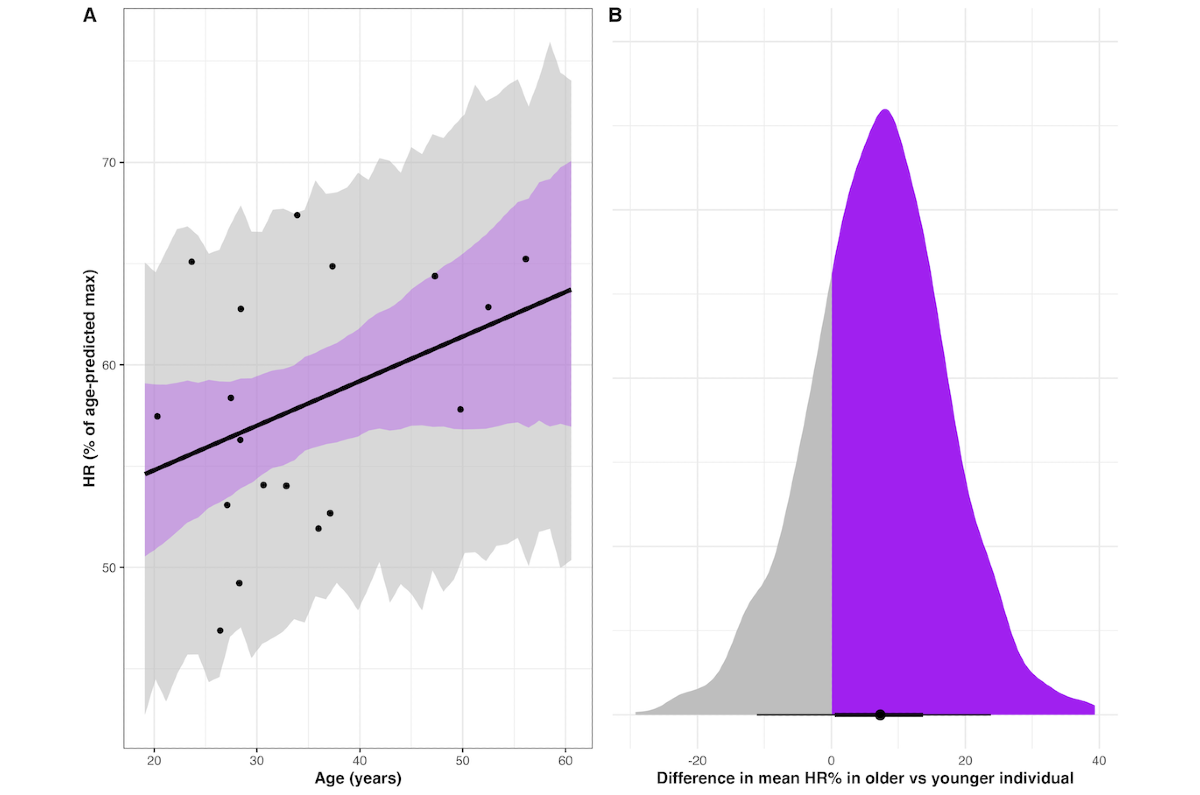
Older participants work at a higher percentage of heart rate max compared to younger participants. (A) Posterior estimates of the relationship between participant age and mean heart rate (HR)% during the CARE protocol. The black line represents the estimated mean regression line, the purple shaded region is the 90% compatibility interval (CI) of the regression line, and the gray shaded area is the 90% CI of the estimated predictions of HR% at each age; the dots represent the raw values for age and mean HR%. (B) The posterior density of the difference in mean HR% in a counterfactual example using 2 hypothetical participants; 1 older (highest sample age, 56) and 1 younger (lowest sample age, 20). The purple shading represents the credible mass of the posterior distribution where there is a greater average exercise HR% in the older individual, while the gray shading shows the credible mass of the posterior distribution where there is a greater average exercise HR% in the younger individual. Zero represents the area in the posterior density where there is no difference in HR% between individuals. The black dot represents the estimated mean difference in HR% between individuals; the thicker and thinner lines represent the 70% and 90% CI’s of the difference, respectively. Plots were comprised from 3000 posterior draws.

On average, older participants worked at a slightly higher HR% compared to younger participants; a difference of ~5 years in age equated to a ~1% increase in HR%, on average. In a counterfactual example using 2 hypothetical individuals, one who is aged 20 years (~lowest age in the current sample) and one who is aged 56 years (~highest age in the current sample), the older individual would have a higher average HR% across the 3 sessions with 79% probability, with an estimated difference in HR% of ~12.2% (90% CI of the difference –13.4% to 38.7%). However, caution is warranted in interpreting the point estimate of the difference given the substantial uncertainty on the posterior contrast. See Figure 3 for a visual depiction of the linear relationship between HR% and age as well as the counterfactual contrast. Additionally, there were no meaningful differences in average HR% across all sessions between users with and without a history of concussion, depression, or headaches, or between those who did or did not follow an exercise program at the time of the study (Figure S2 of Multimedia Appendix 1).

### Symptoms

Prior to the beginning of the exercise plan, participants’ median symptom severity score was 32.5 (IQR 25.8-45.2). Participants also reported whether they felt “the same,” “better” or “worse” following each exercise session. Following session 1, 83% (n=15) of participants reported feeling “the same” or “better,” 72% (n=13) of participants felt “the same” or “better” following session 2, and 76% following session 3 (n=13; of 17 because 1 participant failed to report). Following the 3 sessions, the median symptom severity score was 22 (IQR 19-26), with all but 1 participant reporting a decrease (n=16/17, 94%); the single participant who did not report a decrease had the same symptom score before and after the plan. The average estimated decrease in symptom severity following the protocol was 10 points, although with large uncertainty on the mean estimate (90% CI of the difference 1.1-21.8 points). Hence, while the degree of symptom alleviation varied substantially, completing the 3 sessions lowered symptom severity with 94% probability. The most prominent reductions were observed in cognitive (estimated difference 3 points, 90% CI –1.6 to 7.9 points, 84% of the probability mass >0%) and somatic symptoms (estimated difference 2.7 points, 90% CI –3.7 to 8.8 points, 77.9% probability mass >0%); like total symptom severity, there was large uncertainty on the estimates. See Table 2 for raw symptom scores and estimated paired pre- versus postplan differences, and Figure S3 in Multimedia Appendix 1 for the associated posterior contrasts. Furthermore, while there was no difference in symptom alleviation between participants who did versus those who did not follow an exercise program prior to study enrollment, or in participants who reported versus those who did not report a history of concussion or depression, those with a history of headaches reported less symptom alleviation compared to those without a history of headache; this occurred with 80% posterior probability, but with large uncertainty on the difference estimates (estimated difference 6.9 points, 90% CI –5.8 to 19.5; Figure S4 in Multimedia Appendix 1). However, a symptom reduction was reported following the CARE protocol in both individuals with or without a history of headache (estimated mean symptom reduction=5.1 and 13.2 points, respectively).

**Table 2:** Symptoms before-and-after aerobic exercise plan.

Characteristic	Raw symptom scores			Estimated difference		Participants reporting decrease (N=17), n (%)
	Before (N=18), median (IQR)	After (N=17), median (IQR)	Posterior samples (N=1000), mean (90% compatibility interval)			
Symptom severity	32.5 (25.8-45.2)	22 (19-26)	10 (1.1 to 21.8)		16 (94)	
Somatic	9 (8-13)	8 (5-9)	2.7 (–3.7 to 8.8)		13 (76)	
Cognitive	8.5 (5.2-10.8)	5 (4-6)	3 (–1.6 to 7.9)		14 (82)	
Fatigue	2 (1.2-4)	2 (1-2)	0.3 (–2 to 2.7)		8 (47)	
Sleep	4 (2-5)	3 (0-5)	0.7 (–3.5 to 5.5)		9 (53)	
Neck	1 (1-2)	1 (0-1)	0.4 (–0.9 to 2.1)		7 (41)	
Balance	0 (0-2)	1 (0-1)	0.2 (–1.9 to 2.5)		6 (35)	
Vision	5 (3-6)	3 (1-5)	0.9 (–1.7 to 3.3)		7 (41)	

### Does More Effort Lead to More Symptom Resolution?

There was no relationship between participants’ mean HR% throughout the exercise plan and the amount of symptom alleviation they reported. This held when adjusting for the confound of age (a common cause of symptom reporting and exercise HR%).

## Discussion

### Principal Findings

The findings of our study indicate that it is safe and feasible to provide individuals who are symptomatic from a concussion with an aerobic stimulus delivered through a mobile app. The aerobic stimulus was achieved using primarily bodyweight movements implemented through a CARE protocol, eliciting a relatively consistent HR over 3 sessions. The protocol took approximately 1 week to complete and resulted in meaningful symptom improvement. An important finding from our study was that participants could tolerate the CARE protocol, which was evident by the low number of adverse effects. We wish to highlight that exercise prescriptions for concussion have historically been limited to stationary bikes, treadmills, and other equipment where potential risks to the individual are limited through the careful identification of a target and consistent HR; however, we are encouraged that we found an aerobic stimulus can be delivered through a mobile app using instructional videos that illustrate movement patterns and control work-to-rest time.

Heart rates were either at or below the target range of 55% (±5%) of the age-adjusted max in all but one of the 18 participants. However, that 7 individuals were below the target heart range is not a prominent concern, as one of the primary goals of this study was to understand whether participants could complete the 3-session exercise protocol without adverse effects, which they did. Interestingly, even with 39% (n=7/18) of the sample falling below the target HR range, all but 1 participant reported a decrease in symptoms following the program. This, in concert with our observation that HR did not appreciably correlate with symptom reduction, suggests that program completion and minimal elevation of HR are sufficient to provide symptom relief following concussion. That we observed symptom alleviation in individuals who have been symptomatic for months following their concussion, is also encouraging..

Interestingly, there was not a consistent response to the CARE protocol across all symptom subtypes. We found that symptom reduction was greatest in cognitive, somatic, and vision symptoms, whereas sleep, fatigue, and balance symptoms were relatively unchanged. The interpretation of these results is speculative but provides evidence for future efforts to examine how specific symptom subtypes respond to aerobic activity. There is an emerging body of evidence noting that persistent postconcussion symptoms are a function of a complex combination of preinjury, personality, and injury characteristics [35,36]; for example, patients with persistent, predominantly fatigue symptoms are likely to have a more adaptive personality structure than other patients [35]. The potential clinical relevance of identifying such patients is that they may require a more extended intervention period, or may even be resistant to a single intervention modality of aerobic exercise.

Our findings are consistent with prior research reporting that the use of mobile apps for exercise prescription represents a safe, effective, and feasible approach; this has been illustrated in populations with chronic disease [37,38] and those targeting in-home strength and balance training for older people [39]. Although the benefits of exercise prescription apps are encouraging, including the present study, the variability in attrition rates (5%-62%) is worth noting. In a systematic review of rates of dropout in app-based interventions for chronic disease, the pooled estimate of dropout rates was 43%, with higher rates seen in real-world research and lower rates in highly supported randomized controlled trials [40]. In this study, we observed an attrition rate of 14.3% (n=3). Two of the 3 participants that dropped out of our study were uncomfortable using mobile technology. It is possible that the aid of an introductory video or an offer of periodic one-on-one instructions with research personnel would be helpful. Future studies should qualitatively document and understand reasons for dropout as it will help adoption at scale and over longer periods [40].

### Limitations

Although this study's findings are encouraging, they must be evaluated in the context of a few relevant limitations. First, a small, predominantly female sample size may have inflated the uncertainty of our inferences and potentially reduced the generalizability of our findings to males. However, to combat the former, we used a Bayesian analytical approach with regularizing priors, which allows for relatively stable estimates with smaller amounts of data. Yet, validation in a larger sample with more males is needed. Second, we did not directly observe participants’ exercise sessions, as the protocol was performed through a mobile app delivered to their smartphones. Hence, while HR acquisition allowed us to monitor activity, adherence to the specifics of the exercise protocol cannot be confirmed. Furthermore, there were some issues with HR data acquisition causing us to acquire partial data from 6 of 18 participants (see *Results*). Although this is an expected limitation when conducting studies remotely using wearable devices, this further supports the need for more studies with larger sample sizes to account for data loss. Despite these limitations, however, we were able to show that completing 3 sessions of a CARE protocol with relatively minimal HR elevation led to symptom reduction in all but one participant.

### Conclusion

A CARE protocol delivered through a mobile health platform at 55%-65% of age-adjusted maximum HR can be completed by symptomatic individuals following concussion without adverse effects. The completion of a CARE protocol results in a lower reported symptom burden in almost all cases. The potential for this platform in concussion rehabilitation warrants further investigation.

## Appendix

Multimedia Appendix 1 Supplementary Figure File.

## Abbreviations

CARE: continuous aerobic resistance exercise
CI: compatibility interval
HR: heart rate

## Data Availability

Data and code used to generate the estimates, tables and figures in this manuscript can be found in a public GitHub repository [41].
