# Supplementary material for: A Continuous Aerobic Resistance Exercise Protocol for Concussion Rehabilitation Delivered Remotely via a Mobile App: Feasibility Study
Source: JMIR Form Res. 2023 Jun 19;7:e45321. doi: 10.2196/45321 (PMC10337420; doi:10.2196/45321)
Supplement: Multimedia Appendix 1 [file formative_v7i1e45321_app1.docx]

**Supplementary Figures**

**
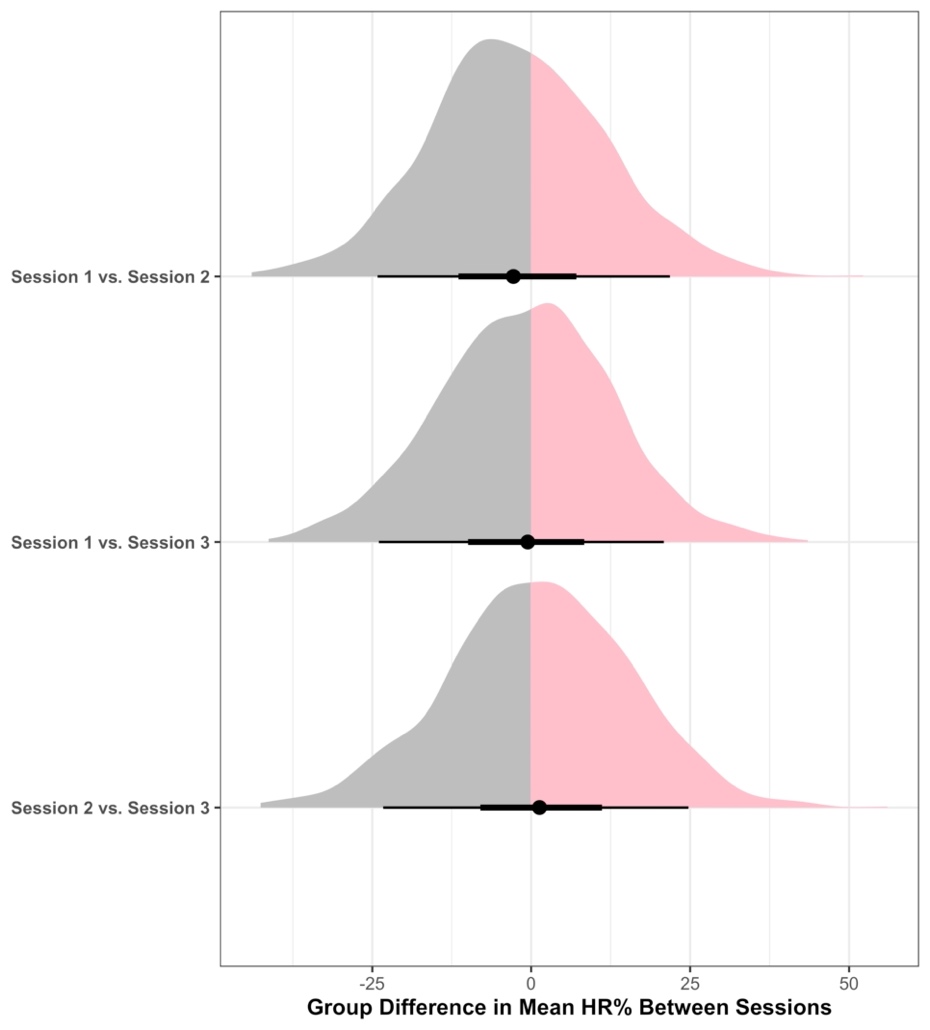
**

*Supplementary Figure 1.* Mean heartrate (HR)% did not differ between sessions. The plots show three contrast densities estimating the difference in HR% between all sessions. The y-axis shows each comparison while the x-axis represents the posterior density of the HR% difference between sessions. The pink shading represents the credible mass of the posterior distribution where there is a higher HR% in the session labelled on the y-axis to the left of the ‘vs’. Conversely, the grey shading represents the credible mass of the posterior distribution where there is a higher HR% in the session labelled on the y-axis to the right of the ‘vs’. Zero represents no difference between sessions. The black dot represents the estimated mean difference in HR% between sessions; the thicker and thinner lines represent the 70% and 90% compatibility intervals of the difference, respectively. Plots were comprised from 2000 posterior draws.


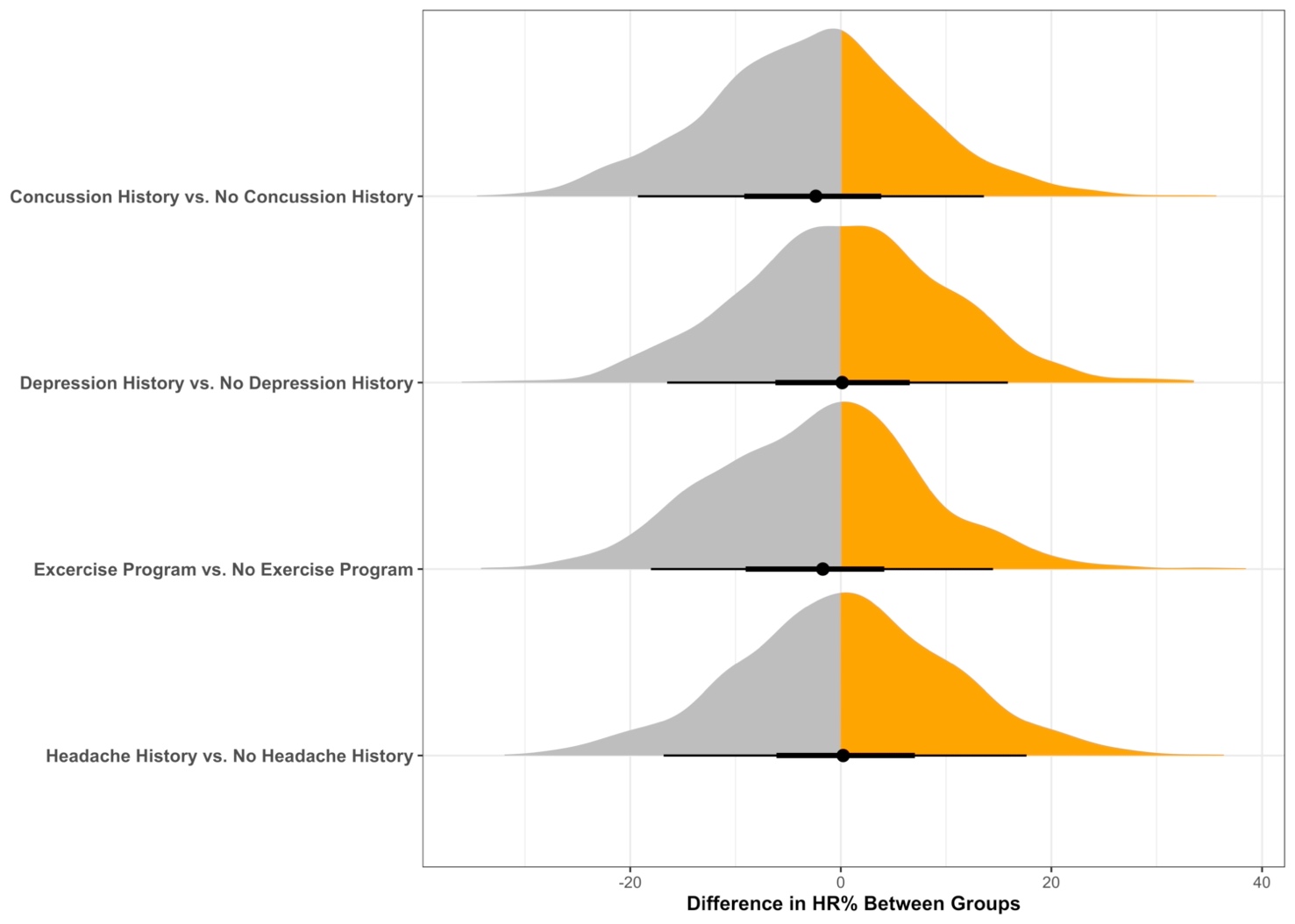


*Supplementary Figure 2.* No group differences in mean HR% across all sessions. The plots show four contrast densities estimating the group differences in mean HR% across all sessions. The y-axis shows each comparison while the x-axis represents the posterior density of the HR% difference between groups. The orange shading represents the credible mass of the posterior distribution where there is a higher HR% in the group labelled on the y-axis to the left of the ‘vs’. Conversely, the grey shading represents the credible mass of the posterior distribution where there is a higher HR% in the group labelled on the y-axis to the right of the ‘vs’. Zero represents no difference between groups. The black dot represents the estimated mean difference in HR% between groups; the thicker and thinner lines represent the 70% and 90% compatibility intervals of the difference, respectively. Plots were comprised from 2000 posterior draws.


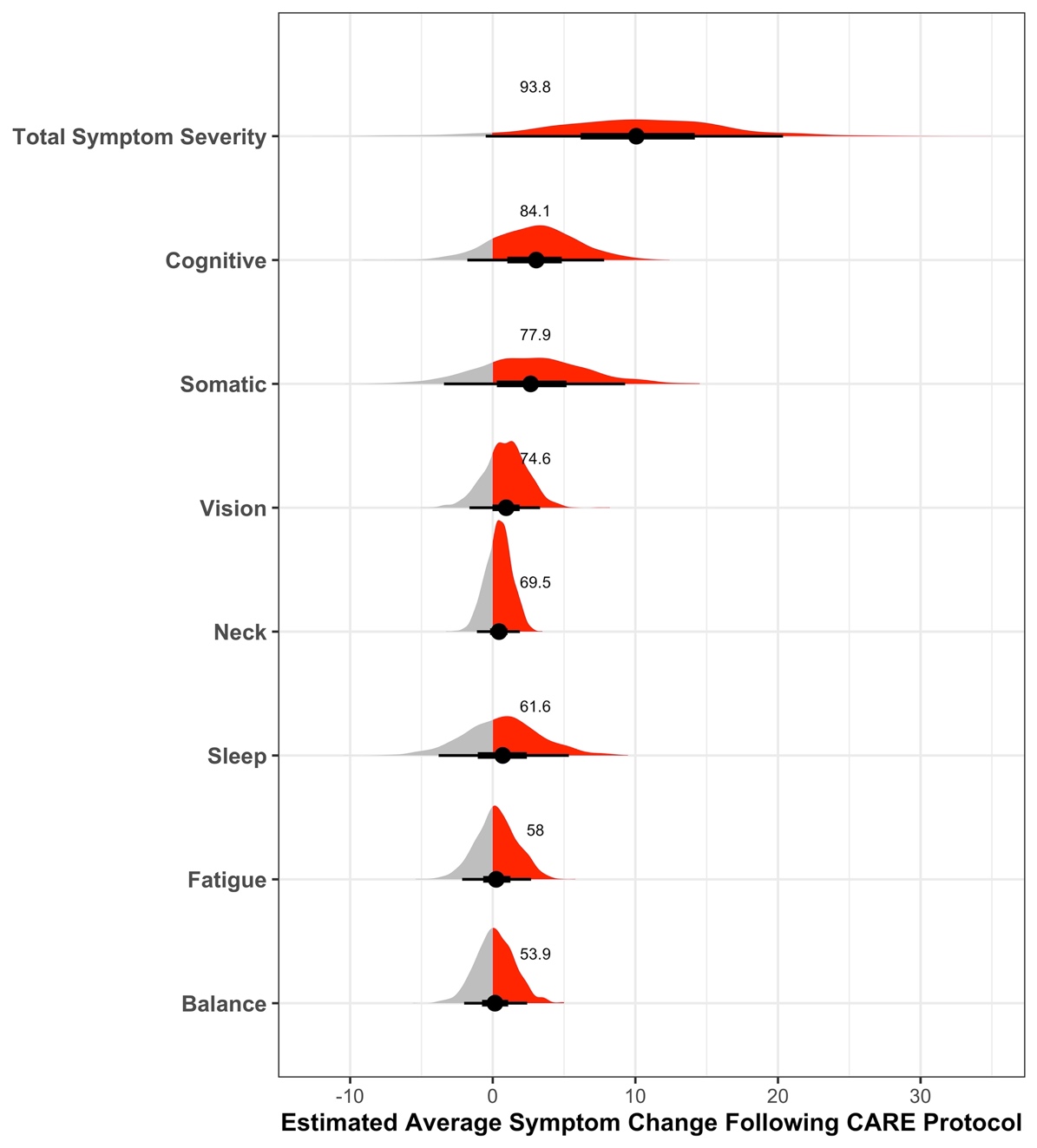


*Supplementary Figure 3.* Symptoms decrease following CARE. The plots show eight contrast densities estimating the difference in symptoms pre vs. post completion of the continuous aerobic resistance exercise (CARE) protocol. The y-axis shows each symptom comparison while the x-axis represents the posterior density of the symptom difference in raw units. The red shading represents the credible mass of the posterior distribution where symptoms decreased following the CARE protocol, while the grey shading represents the credible mass of the posterior distribution where symptoms increased following the CARE protocol. Zero represents no difference in symptoms. The black dot represents the estimated mean difference in symptoms pre vs. post CARE; the thicker and thinner lines represent the 70% and 90% compatibility intervals of the difference, respectively. The numbers next to the densities represent the percentage of the posterior distribution that is shaded red, and therefore the posterior probability that the symptom comparison in question was lower following the CARE protocol. Plots were comprised from 2000 posterior draws.


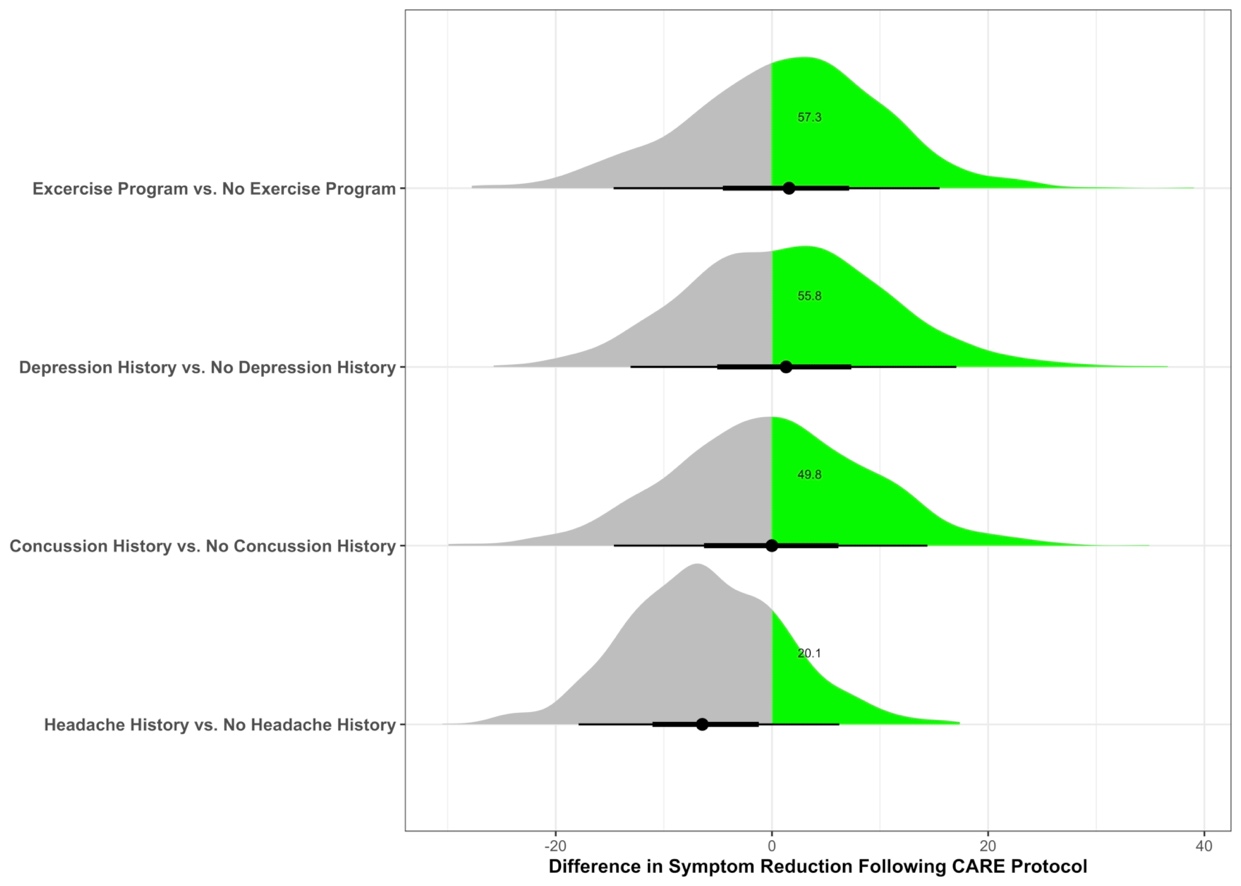


*Supplementary Figure 4.* Estimated group differences in symptom reduction following CARE. The plots show 4 contrast densities estimating the difference in symptoms pre vs. post completion of the continuous aerobic resistance exercise (CARE) protocol. The y-axis shows each symptom comparison while the x-axis represents the posterior density of the symptom difference in raw units. The green shading represents the credible mass of the posterior distribution where there is a higher symptom reduction in the group labelled on the y-axis to the left of the ‘vs’. Conversely, the grey shading represents the credible mass of the posterior distribution where there is a higher symptom reduction in the group labelled on the y-axis to the right of the ‘vs’. Zero represents no difference between groups. The black dot represents the estimated mean difference in symptom reduction between groups; the thicker and thinner lines represent the 70% and 90% compatibility intervals of the difference, respectively. The numbers next to the densities represent the percentage of the posterior distribution that is shaded green

Plots were comprised from 2000 posterior draws.
